# Response of Bovine Cumulus–Oocytes Complexes to Energy Pathway Inhibition during In Vitro Maturation

**DOI:** 10.3390/genes12060838

**Published:** 2021-05-29

**Authors:** Paulina Lipinska, Ewa Sell-Kubiak, Piotr Pawlak, Zofia Eliza Madeja, Ewelina Warzych

**Affiliations:** Department of Genetics and Animal Breeding, Poznan University of Life Sciences, 60-624 Poznan, Poland; paulina.lipinska@up.poznan.pl (P.L.); ewa.sell-kubiak@up.poznan.pl (E.S.-K.); piotr.pawlak@up.poznan.pl (P.P.); zofia.madeja@up.poznan.pl (Z.E.M.)

**Keywords:** oocyte, cumulus cells, energy metabolism, glucose, fatty acids

## Abstract

Glucose or fatty acids (FAs) metabolisms may alter the ovarian follicle environment and thus determine oocyte and the nascent embryo quality. The aim of the experiment was to investigate the effect of selective inhibition of glucose (iodoacetate + DHEA) or FA (etomoxir) metabolism on in vitro maturation (IVM) of bovine COCs (cumulus–oocyte complexes) to investigate oocyte’s development, quality, and energy metabolism. After in vitro fertilization, embryos were cultured to the blastocyst stage. Lipid droplets, metabolome, and lipidome were analyzed in oocytes and cumulus cells. mRNA expression of the selected genes was measured in the cumulus cells. ATP and glutathione relative levels were measured in oocytes. Changes in FA content in the maturation medium were evaluated by mass spectrometry. Our results indicate that only glucose metabolism is substantial to the oocyte during IVM since only glucose inhibition decreased embryo culture efficiency. The most noteworthy differences in the reaction to the applied inhibition systems were observed in cumulus cells. The upregulation of ketone body metabolism in the cumulus cells of the glucose inhibition group suggest possibly failed attempts of cells to switch into lipid consumption. On the contrary, etomoxir treatment of the oocytes did not affect embryo development, probably due to undisturbed metabolism in cumulus cells. Therefore, we suggest that the energy pathways analyzed in this experiment are not interchangeable alternatives in bovine COCs.

## 1. Introduction

Female fertility is a complex trait affected by numerous factors of environmental and physiological origin. Special attention is paid to maternal nutrition, which exerts a significant effect on the follicular environment and metabolic activity of the oocytes [1]. In cows, negative energy balance negatively impacts ovarian follicle growth and oocyte quality [2]. The physiological consequences are characterized by alternations in plasma metabolite levels and further modifications of follicular fluid composition [3]. Experiments revealed a substantial influence of non-esterified fatty acids (NEFAs) concentrations in the follicular fluid (in vivo) or maturation media (in case of the in vitro embryo production systems) on oocyte quality, with a key role of cumulus cells (CCs) in protection against lipotoxicity [4,5]. Cows with fertility problems have significantly lower circulating glucose concentrations, compared to cows with normal fertility [6]. Therefore, an optimal physiological concentration of glucose and fatty acids (FAs) is crucial for proper oocyte development and maturation, which, in the future, may have an impact on embryo developmental potential.

The role of glucose metabolism in bovine cumulus–oocyte complexes (COCs) has been widely described and summarized [7]. The oocyte has a low ability to utilize glucose due to the lack of expression of the specific transporters necessary for glucose uptake. Therefore, cumulus cells support the oocyte by participating in numerous processes including glycolysis, pentose phosphate pathway (PPP), and hexosamine biosynthesis pathway (HBP). Mature COCs consume double the amount of glucose (when compared to immature COCs), which indicates their pivotal role in the maturation process [8]. However, cattle oocytes have been shown to resume meiosis in the absence of exogenous carbohydrates, suggesting a possible reliance on endogenous sources [9]. Experiments that described the effects of glycolysis or PPP inhibition during IVM (in vitro maturation) were mainly focused on mice and pigs and revealed many substantial changes within the COC. In porcine COCs, it resulted in the reduction of glutathione (GSH), adenosine triphosphate (ATP), and maturation promoting factor (MPF) levels in the oocytes. Additionally, inhibition of PPP increased reactive oxygen species (ROS) and reduced the level of nicotinamide adenine dinucleotide phosphate (NADPH). Described modifications during IVM affected the quality of the nascent porcine embryos, where higher apoptosis and cell proliferation rates were observed [10]. Similar effects were described regarding mouse oocytes (delayed meiosis resumption, GSH, and ATP reduction) and embryos (lower blastocyst rate) [11]. The described results clearly demonstrate that limited glucose metabolism does not definitely stop the process of proper oocyte development; however, it induces essential changes, which affect both the oocyte and embryos obtained from such oocytes.

Other important energy sources for the oocytes might be provided in the form of fatty acids, which initially are metabolized via fatty acids oxidation (FAO) in mitochondria. This process is also essential for meiotic progression and oocyte quality determination in many mammalian species [8]. FAs were detected in oocytes, cumulus cells, follicular cells, and in the follicular fluid [12]. FAs are mainly stored in lipid droplets (LDs), which, together with mitochondria, form “metabolic units”. The number of LDs changes significantly during the process of oocyte growth and maturation, and it may also depend on the reproductive status of the female [13,14,15]. Lipid metabolism is essential during the process of bovine oocyte maturation since FAO inhibition reduces significantly the maturation rate as well as disturbs lipid profiling, LDs number, and expression of selected lipid metabolism-related genes in cumulus cells [16].

In the present experiment, three inhibitors affecting the substantial pathways of cell energy production have been applied. Iodoacetate is an inhibitor of glycolysis which forms a thioester bond with the essential cysteine residue in the active center of the glyceraldehyde 3-phosphate dehydrogenase (GAPDH). As a consequence, GAPDH is inactivated and glycolysis is inhibited. DHEA is an inhibitor of glucose-6-phosphate dehydrogenase, the rate-limiting enzyme in PPP. Both inhibitors acting together block the essential part of COC glucose metabolism. In the ETOMOXIR experimental group, the inhibitor etomoxir was used to modify COCs FA metabolism. It is an irreversible inhibitor of the long-chain fatty acid transporter. It works by inhibiting carnitine-palmitoyl-transferase-1 (CPT-1), an enzyme anchored in the outer membrane of the mitochondria, and blocking the entry of long-chain fatty acids into the mitochondria for oxidation.

It has been previously reviewed that both glucose and FAs may be utilized by mammalian oocytes; however, homeostasis between the energy metabolism of lipids and carbohydrates was suggested [17]. It is still unknown what is the extent of the oocyte’s plasticity, and how far the oocyte may redirect its energy metabolism as a response to the external signals (composition of in vitro maturation media or in vivo changes within the follicular environment). Therefore, the aim of our work was to selectively block pathways of either glucose or FAs metabolism at the time of in vitro maturation of bovine COCs to demonstrate how it affects their development, quality, and general energy metabolism. The hypothesis states that the oocyte has the ability to turn its metabolism into a selected energy source (glucose or fatty acids), which is available for the efficient production of energy.

## 2. Materials and Methods

All procedures were performed in accordance with the guidelines of the National Ethics Committee for Animal Research (Ministry of Science and Higher Education, Poland), and no permissions were required.

Unless otherwise stated, all reagents were supplied by Sigma Aldrich.

### 2.1. In Vitro Production of Bovine Embryos

Bovine COCs were collected from slaughterhouse ovaries, selected according to morphology, and in vitro matured for 24 h in the following IVM basic medium: TCM199+Glutamax (Gibco, Thermo Fisher Scientific, MA, USA) supplemented with 6 mg/mL fafBSA, 0.25 mM Na pyruvate, 1x concentrated penicillin–streptomycin solution, 2 µg/mL FSH, 0.02 U/mL LH and 1 µg/mL β-estradiol. The described experiment was conducted on the control group (IVM in basic medium) and two experimental groups: (1) IO+DHEA—IVM medium supplemented with two inhibitors of glucose metabolism [1.5 µM iodoacetate (inhibitor of glycolysis) diluted in water and 150 µM DHEA (inhibitor of pentose phosphate pathway) diluted in DMSO (dimethyl sulfoxide)] and (2) ETOMOXIR—IVM medium supplemented with an inhibitor of fatty acid metabolism (150 µM etomoxir diluted in DMSO).

IVM was performed in four-well plates (Nunc) in 500 µL of maturation medium. For in vitro fertilization (IVF), the semen of two bulls was purchased from the commercial artificial insemination station, and the motile fraction of the sperm was selected with BoviPureTM System according to the producer protocol (Nidacon, Mölndal, Sweden). Shortly, thawed two straws of semen of different bulls were centrifuged in two layers of 40% and 80% BoviPureTM solutions at 300× *g* for 30 min and finally washed in BoviWashTM solution. Gametes coincubation was performed at a final sperm concentration of 1 × 10^6^/mL, and IVF medium was supplemented with PHE (penicillin, hypotaurine, epinephrine). The presumptive zygotes were cultured in groups of 30 in SOF+Na pyruvate medium (Minitube GmbH, Tiefenbach, Germany) supplemented with 0.4 mg/mL fafBSA, 2x concentrated MEM nonessential amino acids, and 0.5x concentrated BME essential amino acids. The culture was performed in 40 μL drops covered with embryo culture tested mineral oil in a humidified atmosphere of 5% CO_2_, 5% O_2_, and 90% N_2_ at 39 °C. On the third day post insemination (dpi), the cleavage rate was estimated, and half of the volume of SOF was replaced with a fresh medium. Embryos were cultured until day 9 (the hatched blastocyst stage in cattle) when blastocyst rate was evaluated (blastocysts number compared to the number of presumptive zygotes). The hatching rate was evaluated by comparing the number of hatched blastocysts to the total number of obtained blastocysts. Hatched blastocyst rate was evaluated by comparing the hatched blastocyst number to the starting number of presumptive zygotes.

### 2.2. ATP Content Analysis in Oocytes

Mature COCs were washed in 0.025% hyaluronidase and pipetted to remove cumulus cells, whereas oocytes were washed in phosphate-buffered saline/polyvinylpyrrolidone (PBS/PVP) and frozen at −80°C in groups of 10. Before analysis, samples were thawed, and oocytes were disrupted in the ultrasonic bath (RT, 3 min). The ATP content was measured by a bioluminescent somatic cell assay kit. All the assay reagents were prepared according to the manufacturer’s instructions. ATP content was quantified by measuring luminescence (Biotek). Measurements were normalized against total protein content in each sample (PierceTM BCA Protein Assay Kit, Thermo Fisher Scientific, MA, USA) and per number of the oocytes in the sample.

### 2.3. Glutathione Content Analysis in Oocytes

COCs after IVM were washed in 0.025% hyaluronidase and pipetted to remove cumulus cells. Oocytes were frozen at −80°C in groups of 25–30.

For the analysis, samples were thawed, and oocytes were disrupted in the ultrasonic bath (RT, 3 min). The total glutathione concentration in oocytes was measured by colorimetric assay using the glutathione assay kit according to the protocol described by [18]. Measurements were normalized against total protein content in each sample (PierceTM BCA Protein Assay Kit, Thermo Fisher Scientific, MA, USA) and per number of the oocytes in the sample.

### 2.4. Lipid Droplets Staining in Oocytes and Cumulus Cells

COCs collected for LD analysis were washed in 0.2% PBS/PVP, and then cumulus cells were separated from oocytes by vigorous pipetting. Oocytes were fixed in 500 µL of 4% paraformaldehyde (PFA) and stored at 4 °C for further analysis. Cumulus cells were placed on adhesive slides (SuperFrost Menzel, Thermo Fisher Scientific, MA, USA), fixed in 4% PFA in a Coplin jar, and stored at 4 °C in 0.2% PBS/PVP for further analysis.

Oocytes were permeabilized in 0.2% Triton X-100 solution for 30 min at RT and washed in 0.2% PVP/PBS. The fluorescent dye used to stain lipid droplets was 20 μg/mL BODIPY 493/503 (Thermo Fisher Scientific, MA, USA). Incubation was performed in 500 μL of the dye solution in PBS at room temperature for 1 h. The chromatin of the nucleus and the polar body were visualized by staining the oocytes with 0.5 μg/mL 4′,6-diamidino-2-phenylindole (DAPI; Vector Laboratories, Burlingame, CA, USA). Oocytes were mounted on a glass slide with a single concave (Comex, Wroclaw, Poland), covered, and stored at 4 °C.

Cumulus cells were stained with the same protocol as for the oocytes. Permeabilizing and washing steps were conducted in Coplin jars, whereas 500 µL of dye solution was transferred directly on the slide and covered with a plastic cover slip to avoid evaporation.

Oocytes and cumulus cells were analyzed using a confocal microscope Zeiss LSM 880 using a 488 nm filter with bandpass 500–550 nm for BODIPY 493/503 (Laser Argon2, Lasos, Jena, Germany) and 420–480 nm for DAPI (Laser Diode 405) according to the previously described methodology [19]. Obtained images were analyzed with the ImageJ Fiji software (NIH, Bethesda, MD, USA). Total lipid content was received by establishing integrated density, oocyte area, and average background parameters in ImageJ Fiji and calculated according to the formula: [Integrated density − (average background × oocyte area)], where integrated density is the sum of values of the pixels in the specified area, and average background is the mean value of fluorescence of the area outside the examined structure. Calculation of the LD size and LD count parameters was obtained by assigning the ImageJ Fiji software. Particles not smaller than 0.2 µM were included in the analysis. In order to calculate the number of single droplets, the “watershed” function was used, and larger aggregates of droplets were mechanically separated.

### 2.5. Quantitative Gene Expression Analysis in Cumulus Cells

Each sample of cumulus cells originated from a group of 30 COCs matured together in one well. After in vitro maturation, COCs were transferred to PBS/PVP, pipetted, and separated; oocytes were removed. Cumulus cells were collected in a tube, centrifuged (1000× *g* for 5 min), the supernatant was removed, and cells were frozen at −196 °C.

The total RNA was extracted with a High Pure miRNA Isolation Kit (Roche, Basel, Switzerland) according to the manufacturer’s protocol. The RNA was precipitated with NF Pellet Paint Co-Precipitant. A total of 1 μL of Pellet Paint, 10 μL of 3 M sodium acetate, and 200 μL of 96% ethanol were added to the RNA sample. After 5 min incubation, the samples were centrifuged at 18000 rcf for 10 min. The RNA pellet was washed and centrifuged in 75 and 96% ethanol and dried at 40 °C. RNA was resuspended in 8 μL of water and measured with Nanodrop 2000c (Thermo Fisher Scientific) to evaluate the OD260/280 ratio value. Further, reverse transcription was applied on total isolated RNA using Transcriptor First Strand cDNA Synthesis Kit (Roche, Warsaw, Poland) following the manufacturer’s protocol. The cDNA samples were diluted 1:1 in water and stored at −20 °C until further analysis. Genes responsible for fatty acid metabolism (*ACACA, FASN, PLIN2, CD36, CPT1B*), glucose metabolism (*G6PDH, PDHA1, PFKP, SLC2A1, CS, LDHA*), and oxidative stress protection (*TXNRD1*) were analyzed. Each cDNA sample was analyzed in two independent PCR runs, and the mean value was used for the calculation of relative transcript abundance to the geometrical mean of reference genes *GAPDH* and *YWHAZ*. Reference genes were estimated with the Normfinder algorithm and the following stability values were obtained: 0.001 for GAPDH and 0.000 for YWHAZ, indicating those genes as the best choice. The lists of analyzed genes, primer, and probe sequences designed by TIBMolbiol (TIB Molbiol Syntheselabor GmgH, Berlin, Germany) are shown in Table 1. Their complementarity to the indicated genes has been analyzed (Primer-BLAST analysis) and the results are shown in the Supplementary File S1. Real-time PCR was performed using the standard curve method. For this purpose, all analyzed genes were first amplified by PCR and visualized on 1.5% agarose gel. The PCR product was isolated and purified using the GeneJet Gel Extraction Kit (Thermo Fisher Scientific). DNA concentration was measured with a Nanodrop 2000c (Thermo Fisher Scientific) and serial tenfold dilutions of DNA with a known concentration (standards) were generated. The standards were used in a real-time PCR reaction to produce the appropriate standard curve with the LightCycler 480 II software (Roche). The reactions were performed using the LightCycler 480 II system with a set of supplied reagents (LightCycler 480 Probes Master, Roche). The 10 μL reaction mixture consisted of 5 μL of the 2 × concentrated LightCycler Probe Master, 0.5 μM primers, 0.3 μM probe, and 1 μL of cDNA. The following reaction conditions were applied: denaturation at 95 °C for 10 min; amplification (40 cycles) at 95 °C for 10 s, 58 °C for 30 s, and 72 °C for 1 s; and final cooling at 40 °C.

### 2.6. Mass Spectrometry—Samples Preparation

For mass spectrometry analysis, each COC was matured separately in micro-Insert 4 Well FulTrac plates (ibidi) in 10 µL of medium per each microwell covered with oil. As a result of this approach, from each microwell, a set of separate samples was collected—oocyte, cumulus cells, and maturation medium.

After IVM, each COC, together with the maturation medium, was removed from the microwell and placed on a new plate; then, the COC was removed to the fresh drop of PBS, whereas the maturation medium was immediately frozen in liquid nitrogen. The COC was pipetted vigorously in PBS drop to separate cumulus cells from the oocyte; the oocyte was moved to the fresh drop of PBS, while cumulus cells were frozen in liquid nitrogen. The oocyte was then incubated in hyaluronidase (0.025%) and pipetted vigorously, it was washed twice in PBS and frozen in liquid nitrogen. Since the analysis of the maturation medium aimed at investigating the consumption of metabolites and lipids during IVM, an additional sample of 10 µL of the freshly prepared pre-IVM medium was frozen.

### 2.7. Mass Spectrometry—Metabolomic Analysis

Samples were treated with 100 µL of cold 80% methanol, and metabolite extraction was performed by sonication for 10 min. Samples were then vortexed for another 5 min and centrifuged at 11,000× *g* for 5 min. Next, supernatants were transferred to new Eppendorf tubes. Methanol extracts were evaporated using a vacuum concentrator (Labconco, Kansas City, MO, USA); samples were then additionally dried out under vacuum over P_2_O_5_ and derivatized to block polar groups of compounds present in the mixture. The derivatization was carried out by incubation for 1.5 h at 37 °C with 80 µL of 20 mg/mL methoxyamine hydrochloride in pyridine, followed by incubation with 100 µL of *N*-methyl-*N*-(trimethylsilyl)trifluoroacetamide (MSTFA) for 30 min at 37 °C.

Qualitative and quantitative assays of the samples were performed using a LECO Pegasus 4D system consisting of a 7890A gas chromatograph (Agilent, Santa Clara, CA, USA) and LECO ToF mass analyzer. Data were acquired and initially analyzed using LECO ChromaTOF software version 4.51.6.0. Gas chromatography was performed using a 30 m long, 0.25 mm internal diameter DB-5MS column with 0.25 µM film thickness (J&W Scientific, Agilent). For injection, a Gerstel CIS PTV-type injector was used. The injection temperature was 40 °C running up to 240 °C (10 °C/s), the MS transfer line was set to 250 °C, and the ion source was adjusted also to 250 °C. Pure helium was used as the carrier gas at a constant flow of 1 mL/min. The oven temperature was held constant at 70 °C for 2 min, then ramped at 10 °C/min to 300 °C, and finally held constant for 10 min at 300 °C. Mass spectra were recorded in a range of 50–850 m/z in EI + mode under standard 70 eV ionization conditions. The retention index mixture was run prior to relevant analyses.

Raw data files were exported to netCDF format and then converted to abf format for analysis using MSDial software (v. 3.96). To eliminate the retention time (Rt) shift and to determine the retention indexes (RI) for each compound, the alkane series mixture (C-10 to C-36) correction was implemented. For compound identification, the database from the CompMS site containing 28,220 records was used. Identified artifacts (alkanes, column bleed, plasticizers, MSTFA, and reagents) were excluded from further analyses. Obtained normalized (using total ion current (TIC) approach) results were then exported to Excel for preformatting and then used for statistical analyses.

### 2.8. Mass Spectrometry—Lipidomic Analysis

Lipid separation was carried out according to modified MTBE extraction protocol by [20]. The whole sample was put in glass tubes with Teflon-coated caps. Next, 150 μL of methanol and 0.5 mL of MTBE were added and mixtures were vortexed for 1 h at RT. Phase separation was induced by the addition of 125 μL of water. Upon 10 min incubation at RT, mixtures were centrifuged at 1000× *g* for 10 min and 0.2 mL of upper organic phase were then collected and dried under a nitrogen stream at 37 °C. Samples were directly resuspended prior to analysis in 100 μL of MS-mix buffer containing 7.5 mM ammonium acetate in chloroform, 2-propanol, and methanol (1:2:4 *v*/*v*/*v*).

Lipid profiling was performed using Q-Exactive Orbitrap mass spectrometer (Thermo Fisher Scientific, Bremen, Germany) equipped with TriVersa NanoMate nanoflow ESI ion source (Advion BioSciences ltd., Ithaca, NY, USA). For this, 10 μL sample aliquots were infused directly into the mass spectrometer, and after ion current stabilization, samples were measured for 10 min. The source was operated at a gas pressure of 1.25 psi, and the ionization voltage was set to 1.05 kV. MS data were acquired in positive ion mode within the range of *m/z* 300–1500 at the resolution of 140,000 (at *m/z* 200, Full Width at Half Maximum, FWHM). Automatic gain control was set to a target value of 3 × 106, and ion injection time (IT) was 100 ms.

MS data (RAW files) were converted into mzXML format and further processed using LipidXplorer software ver. 1.2.8.1, developed at Max Planck Institute of Cell Biology and Genetics in Dresden (Germany) [21]. Profiles were obtained by averaging 8 min of recorded mass spectra, from the second minute to the ninth minute within ten minutes of sample delivery time, while the first 60 s of sample injection were allowed for electrospray and analyte flow stabilization observed based on total ion current (TIC) variation. Subsequently, alignment was performed in order to match related peaks within the whole dataset. Defined signals in mass spectra for further processing and consideration were selected by setting the value of the threshold to 1500. Particular lipid species were identified afterward based on accurately determined masses related to mass accuracy better than 5 ppm. Data were then exported to text format and opened in Microsoft Office Excel software (ver. 2010) for preformatting purposes. Intensities of each lipid peak were normalized to TIC, and the table was formatted in order to be loaded to statistical software.

### 2.9. Statistical Analysis

The evaluation of the distribution of collected data was carried out by Shapiro–Wilk test in statistical package R (https://cran.r-project.org/, accessed on 15 September 2020). In most cases, the Shapiro–Wilk test indicated the nonparametric distribution of the data. If the Shapiro–Wilk indicated normal distribution, then also skewness and kurtosis of the parameters were evaluated. In all cases, this additional evaluation would indicate either strong skewness and/or kurtosis. Thus, the decision was made to use a nonparametric Kruskal–Wallis rank-sum test [22] for further statistical analysis of the data. To obtain multiple pairwise comparisons between all experimental groups after the Kruskal–Wallis test, Dunn’s test [23] was applied. All analyses were performed with statistical package R (https://cran.r-project.org/). 

Values were estimated as significantly different when *p*-value was lower than 0.05 and highly significantly different when the *p*-value was lower than 0.01.

Metabolomic analysis (enrichment analysis) was conducted through MetaboAnalyst 5.0 software.

## 3. Results

### 3.1. IVP Efficiency

**Figure 1:**
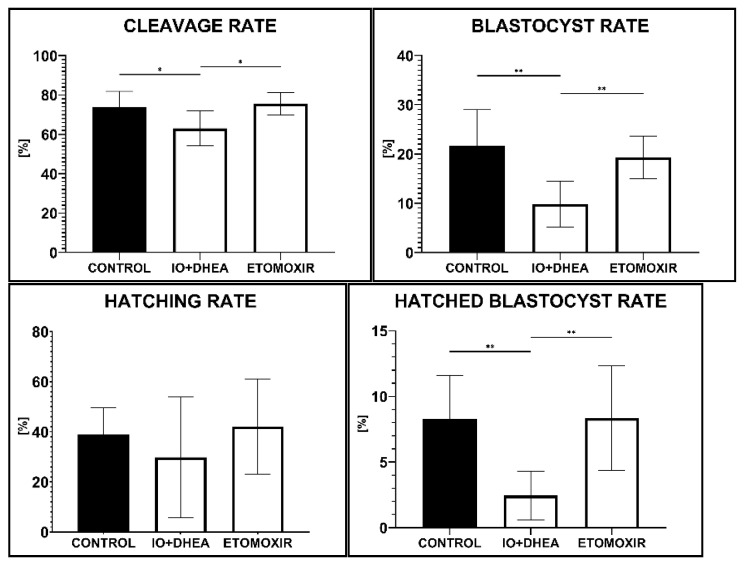
The effect of glucose (IO+DHEA) and fatty acid (ETOMOXIR) metabolism inhibition on the efficiency of in vitro embryo production (means ± SD). *—*p* ≤ 0.05, **—*p* ≤ 0.01.

Supplementation of maturation medium with glucose or fatty acid metabolism inhibitors affected the efficiency of in vitro embryo production. Surprisingly, inhibition of FAO (ETOMOXIR experimental group) changed neither cleavage nor blastocyst rate. However, when glucose metabolism was inhibited (IO+DHEA experimental group), a significant decrease in cleavage, blastocyst rate, and hatched blastocyst rate was observed (Figure 1).

### 3.2. Glutathione Content

**Figure 2:**
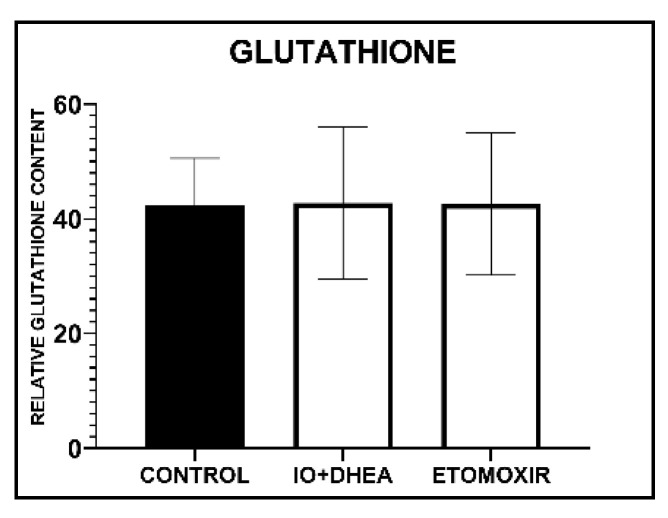
The effect of glucose (IO+DHEA) and fatty acid (ETOMOXIR) metabolism inhibition on glutathione content in mature oocytes (means ± SD).

The relative concentration of glutathione was analyzed in 23 samples (control, *n* = 7; 8 IO+DHEA, *n* = 8; ETOMOXIR, *n* = 8). Each sample consisted of 25–30 pooled oocytes. Statistical analysis showed no effect of IO+DHEA and ETOMOXIR treatment on glutathione content in oocytes (Figure 2).

### 3.3. ATP Content

**Figure 3:**
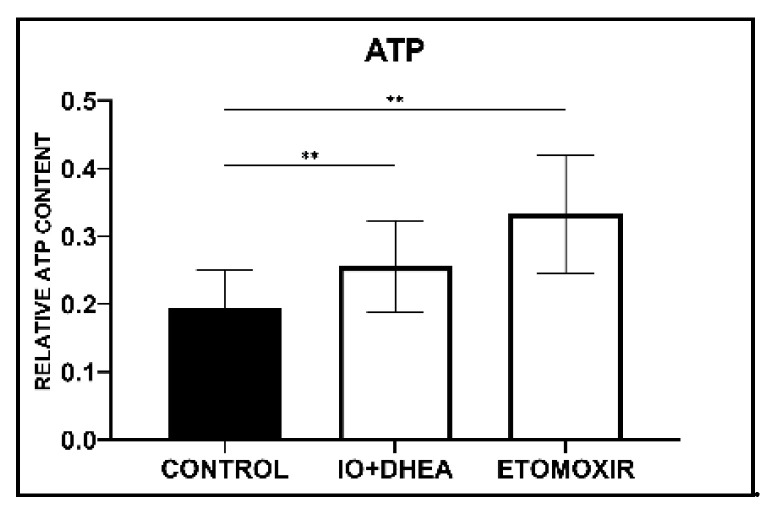
The effect of glucose (IO+DHEA) and fatty acid (ETOMOXIR) metabolism inhibition on ATP content in oocytes (means ± SD). **—*p* ≤ 0.01.

ATP relative concentration was analyzed in 31 samples (control, *n* = 11; IO+DHEA, *n* = 10; ETOMOXIR, *n* = 10). Each sample consisted of 10 pooled oocytes. It was shown that both glucose and fatty acids metabolism inhibition during IVM significantly increased ATP content in oocytes when compared to control (Figure 3).

### 3.4. Lipid Droplet Staining in Oocytes and Cumulus Cells

**Figure 4:**
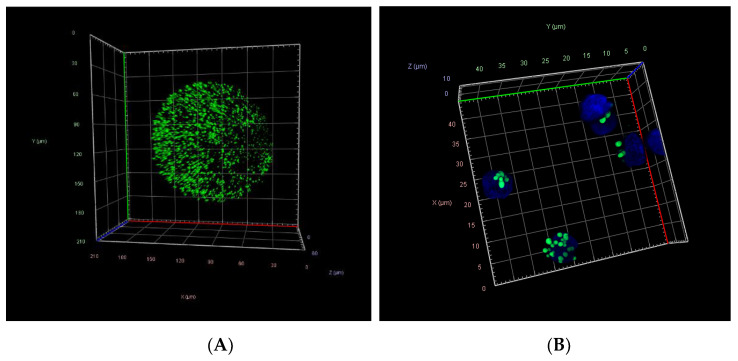
Confocal 3D projections of fluorescently stained oocytes (**A**) and cumulus cells (**B**) with BODIPY 493/503 (green—lipid droplets) and 4′,6-diamidino-2-phenylindole (DAPI; blue—nuclei).

**Figure 5:**
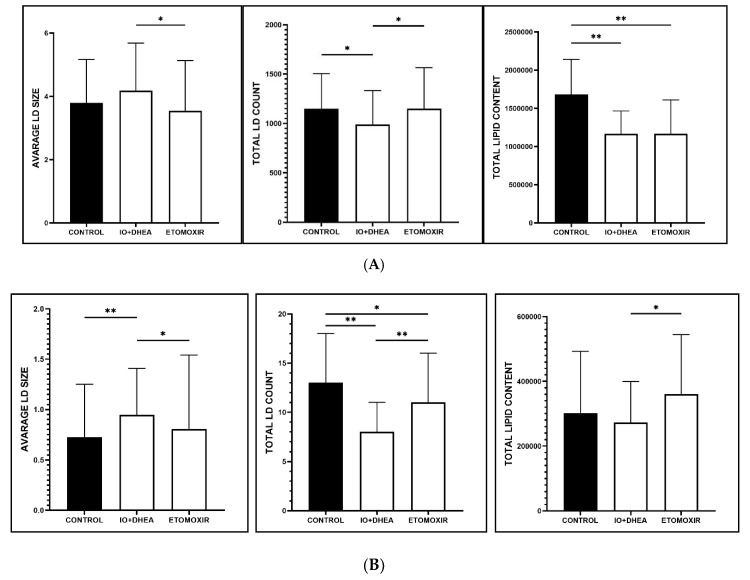
The effect of glucose (IO+DHEA) and fatty acid (ETOMOXIR) metabolism inhibition on the lipid droplets parameters in (**A**) oocytes and (**B**) cumulus cells (means ± SD). *—*p* ≤ 0.05, **—*p* ≤ 0.01.

Lipid droplets were analyzed in 123 individual oocytes (control, *n* = 40; IO+DHEA, *n* = 44; ETOMOXIR *n* = 39; Figure 4A). Regarding cumulus cells, 109 samples of CC representing individual COCs were analyzed (control, *n* = 41; IO+DHEA, *n* = 39; ETOMOXIR *n* = 29; Figure 4B). Confocal microscopy imaging revealed several significant alternations in oocytes and cumulus cells in response to the two inhibitor systems used. As shown in Figure 5, total lipid content decreased in oocytes but did not change in cumulus cells in both IO+DHEA and ETOMOXIR experimental groups. LD count was reduced in all groups of oocytes and CC except for oocytes matured with etomoxir. Regarding the size of LD, this parameter was not affected by etomoxir neither in oocytes nor in CC; however, it increased in cumulus cells of the IO+DHEA group.

### 3.5. mRNA Expression in Cumulus Cells

**Figure 6:**
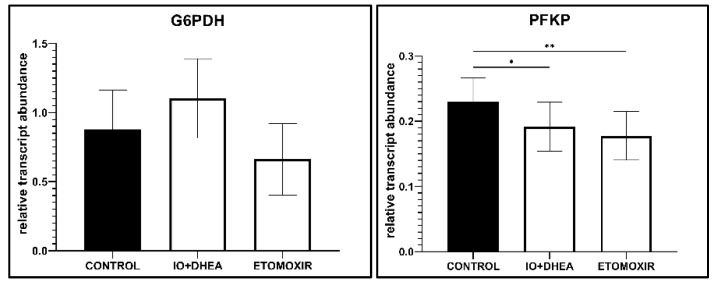

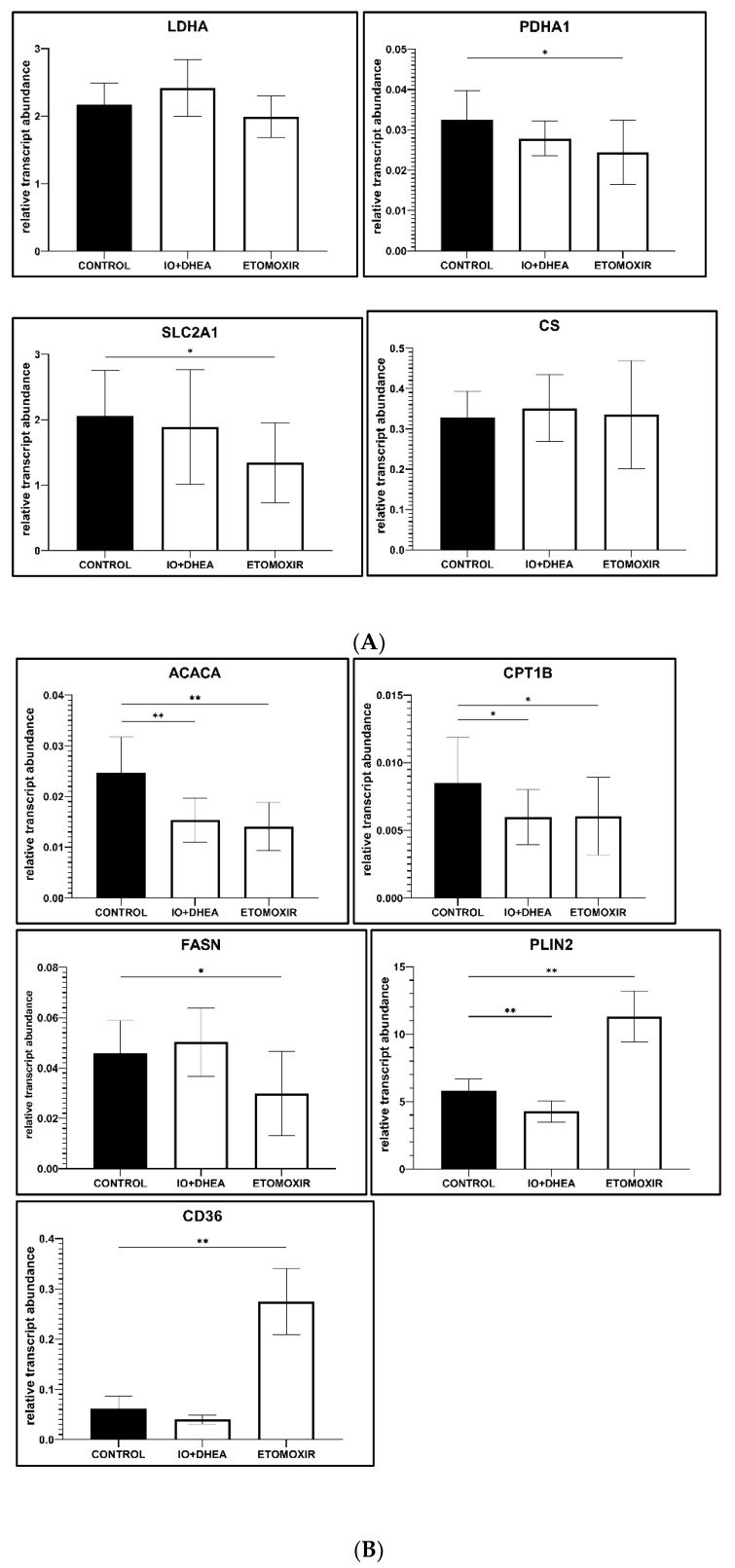

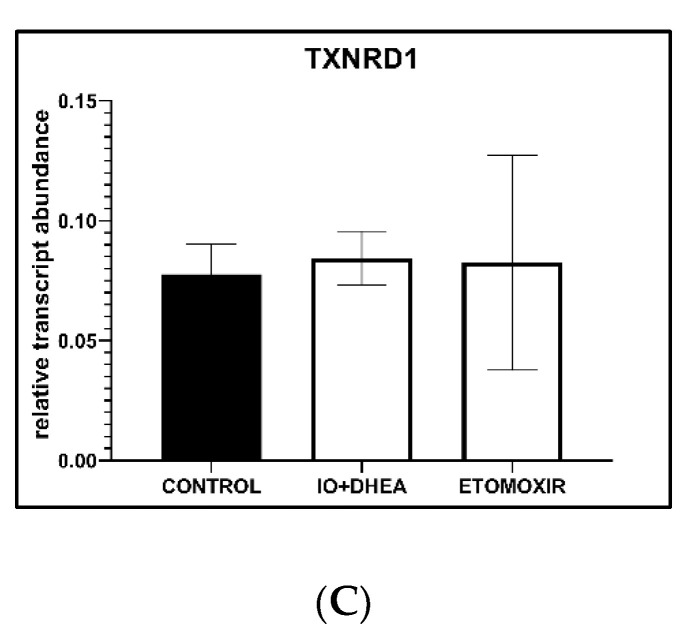
The effect of glucose (IO+DHEA) and fatty acid (ETOMOXIR) metabolism inhibition on the selected gene expression levels (mRNA) in the cumulus cells. The results are shown as a transcript abundance relative to the geometric mean of reference genes (means ± SEM). *—*p* ≤ 0.05, **—*p* ≤ 0.01: (**A**) genes involved in glucose metabolism; (**B**) genes involved in fatty acid metabolism; (**C**) genes involved in oxidative stress protection.

Transcripts level in CC was analyzed in 28 samples (control *n* = 10; IO+DHEA, *n* = 8; ETOMOXIR, *n* = 10). Significant changes in the mRNA level of several genes involved in energy metabolism were detected. When compared to control, glucose metabolism inhibitors altered the transcript level of four genes (*PFKP*, *ACACA*, *PLIN*, and *CPT1B*). The inhibition of FAO with etomoxir resulted in eight differentially expressed genes (*PFKP*, *PDHA1*, *SLC2A1*, *ACACA*, *PLIN2*, *CPT1B, FASN*, and *CD36*; Figure 6).

### 3.6. Metabolome Analysis in Oocytes and Cumulus Cells

**Figure 7:**
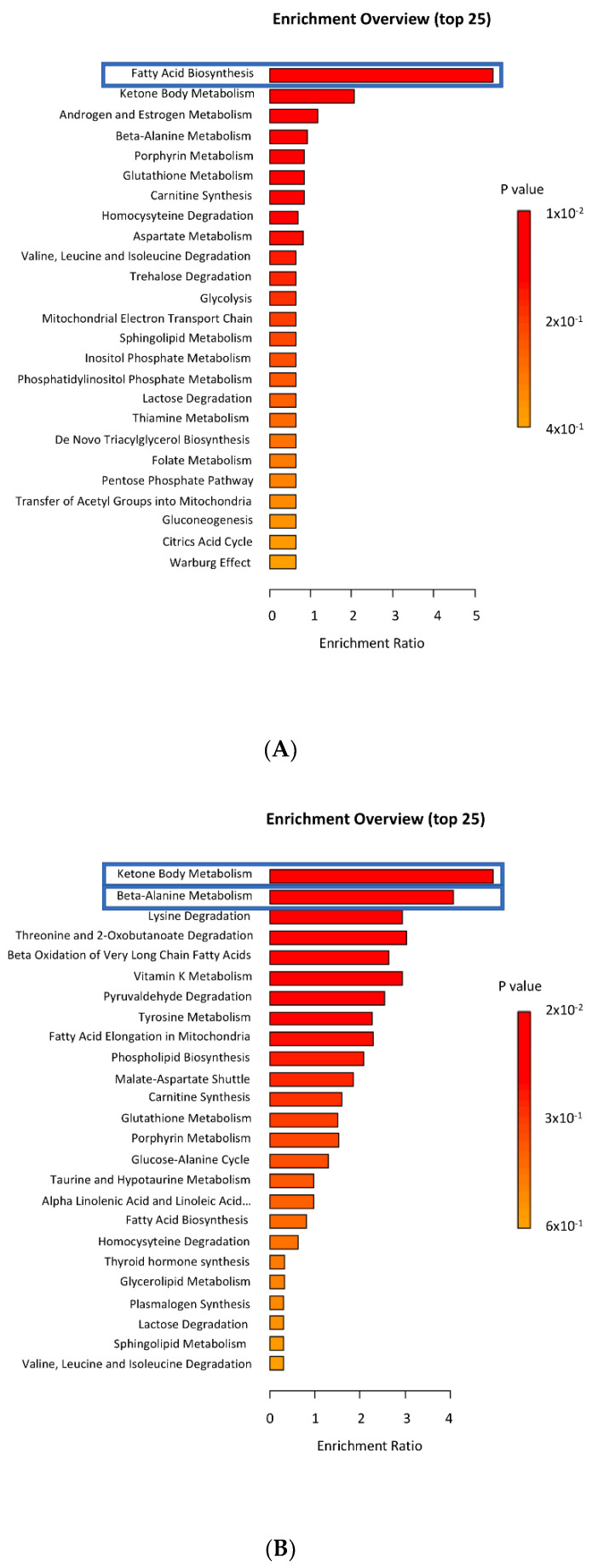

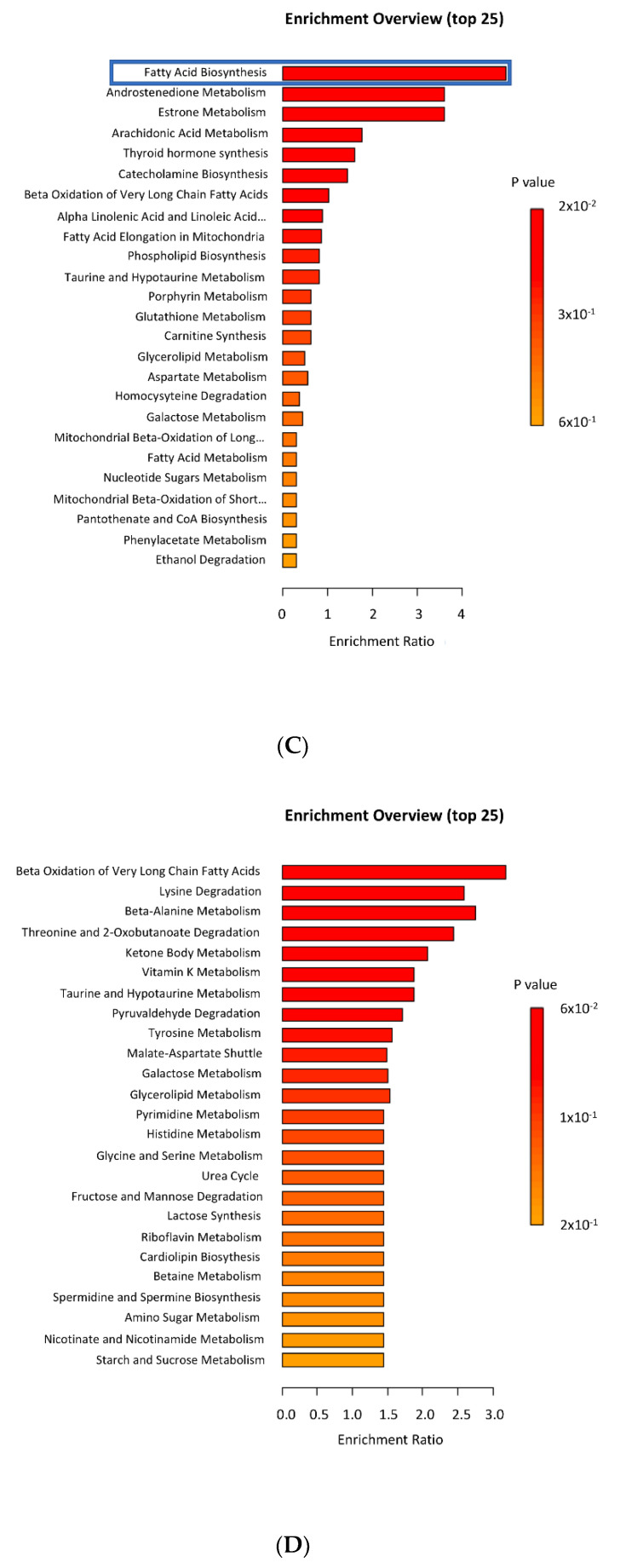
Enrichment metabolites analysis in oocytes and cumulus cells matured with IO+DHEA or ETOMOXIR inhibitors (data compared to control). Pathways that differed significantly (*p* ≤ 0.05) were marked with blue squares: (**A**) control vs. IO+DHEA in oocytes; (**B**) control vs. IO+DHEA in cumulus cells; (**C**) control vs. ETOMOXIR in oocytes; (**D**) control vs. ETOMOXIR in cumulus cells.

The GC/MS allowed the identification of 157 metabolites present in oocytes and cumulus cells. Based on the MetaboAnalyst 5.0 software analyses, the enrichment metabolomic data were compared between the control and the IO+DHEA experimental group as well as between the control and the ETOMOXIR group. Both procedures were applied to study changes in the oocytes and the cumulus cells. The analyses showed that glucose metabolism inhibition significantly downregulated the fatty acid biosynthesis pathway in oocytes and upregulated ketone body metabolism as well as β-alanine metabolism in the cumulus cells. Similarly, inhibition of FAO during IVM also significantly downregulated fatty acid biosynthesis, whereas it did not induce any substantial changes to the activity of the metabolic pathways present in the cumulus cells (Figure 7).

Comparison of control vs. IO+DHEA oocytes showed significant downregulation of fatty acid biosynthesis pathway (*p* = 0.015). The following metabolites within this pathway were identified: palmitic acid, 3-hydroxybutyric acid, caprylic acid, capric acid, dodecanoic acid, malonic acid, myristic acid. Comparison of control vs. IO+DHEA cumulus cells showed significant upregulation of ketone body metabolism (*p* = 0.022). Succinic acid was identified within this pathway. Additionally, significant upregulation of β-alanine metabolism in IO+DHEA cumulus cells was detected (*p* = 0.038). The following metabolites within this pathway were identified: β-alanine, L-glutamic acid, L-aspartic acid, oxoglutaric acid.

Comparison of control vs. ETOMOXIR oocytes showed significant downregulation of fatty acid biosynthesis pathway (*p* = 0.02). The following metabolites within this pathway were identified: palmitic acid, 3-hydroxybutyric acid, caprylic acid, capric acid, dodecanoic acid, malonic acid, myristic acid. There were observed no significant differences between control and ETOMOXIR groups in cumulus cells.

### 3.7. Lipidome Analysis in Oocytes and Cumulus Cells

**Figure 8:**
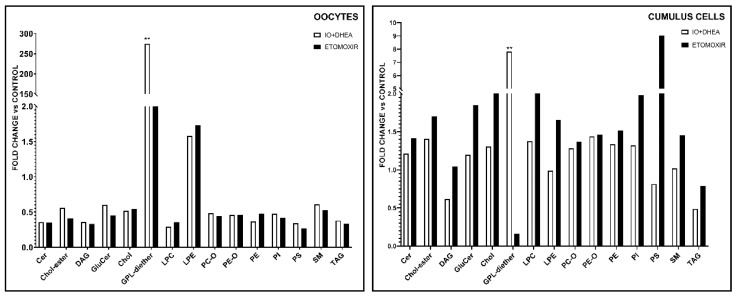
The effect of glucose (IO+DHEA) and fatty acid (ETOMOXIR) metabolism inhibition on the lipidome in oocytes and cumulus cells. The results are shown as a fold change relative to the control group (means of experimental groups divided by means of control group). **—*p* ≤ 0.01. Cer (ceramides); Chol-ester (cholesterylester); DAG (diacylglycerols); GluCer (glucosylceramide); Chol (cholfragment); LPC (lysoPhosphatidylcholine); LPE (lysoPhosphatidylethanolamine); PC-O (phosphatidylcholineether); PE-O (phosphatidylethanolamineether); PE (phosphatidylethanolamine); PI (phosphatidylinositol); PS (phosphatidylserine); SM (sphingomyelin); TAG (triacylglycerol).

**Figure 9:**
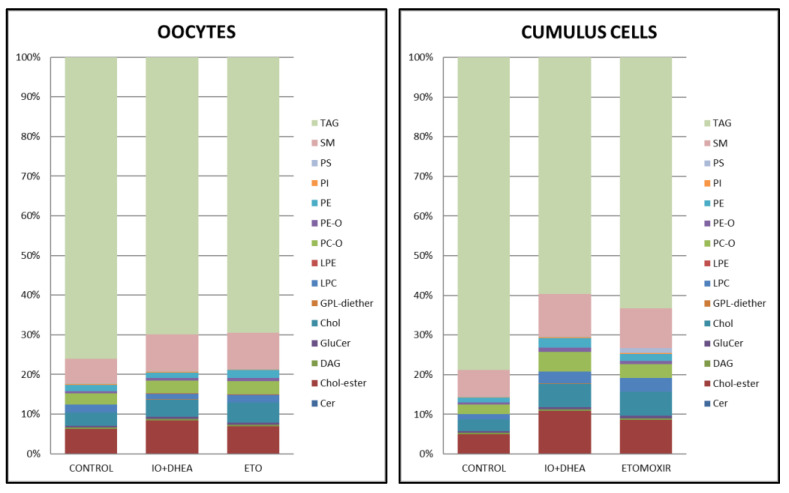
The distribution of lipid classes regarding experimental group (CON vs. IO+DHEA vs. ETO) or cell type (OOCYTES vs. CUMULUS CELLS). Cer (ceramides); Chol-ester (cholesterylester); DAG (diacylglycerols); GluCer (glucosylceramide); Chol (cholfragment); LPC (lysoPhosphatidylcholine); LPE (lysoPhosphatidylethanolamine); PC-O (phosphatidylcholineether); PE-O (phosphatidylethanolamineether); PE (phosphatidylethanolamine); PI (phosphatidylinositol); PS (phosphatidylserine); SM (sphingomyelin); TAG (triacylglycerol).

Lipidome analysis allowed the quantification of 344 lipids from 15 major classes as follows: 25 lipids belonging to ceramides (Cer), 21 to cholesterylesters (CEs), 50 to diacylglycerols (DAGs), 17 to glucosylceramides (GluCers), 1 to glycopeptidolipid (GPL) diether, 16 to lysophosphatidylcholines (LPCs), 18 to lysophosphatidylethanolamines (LPEs), 26 to phosphatidylcholineethers (PC-O), 25 to phosphatidylethanolamineethers (PE-O), 30 to phosphatidylethanolamines (PEs), 16 to phosphatidylinositols (PIs), 13 to phosphatidylserines (PSs), 27 to sphingomyelins (SMs), 58 to triacylglycerols (TAGs), and free cholesterol (Chol). Comparison of the major classes of lipids revealed that only GPL diether significantly differed between control and IO+DHEA experimental group (*p* < 0.01), both in oocytes and cumulus cells (IO+DHEA/CON rate—274 and 7.8, respectively, Figure 8). Other lipid classes did not differ due to high sample variability. Moreover, the TAGs were shown to be the dominant class of lipids both in oocytes and cumulus cells. The distribution of lipid classes regarding the experimental group (CON vs. IO+DHEA vs. ETO) or cell type (OOCYTES vs. CUMULUS CELLS) is presented in Figure 9.

### 3.8. Fatty Acids Uptake/Secretion in Maturation Medium

The metabolome composition of the maturation medium, collected after maturation of single COC (post IVM medium), was compared to the fresh maturation medium collected before IVM (pre IVM medium). It allowed showing whether a single COC secreted free FA to the medium (Table 2, values shown in red) or whether COC absorbed FA from the medium during IVM (Table 2, values shown in green). When results obtained for the experimental groups were compared to the control, the type of change (uptake or secretion) and its relation to the control could be characterized. Data showed that IO+DHEA COCs absorbed three FAs and secreted seven FAs during IVM to maturation medium; however, only pentadecanoic acid secretion differed from control. Interestingly, ETOMOXIR COCs absorbed five FAs and secreted also five FAs during IVM, and most of those observations were significantly different from the control.

## 4. Discussion

The presented study was intended to verify the hypothesis of the existence of possible homeostasis between the two main cellular pathways of energy production—glucose or fatty acids—in bovine oocytes. The obtained results indicate that glucose (not the fatty acids) metabolism during oocyte IVM is substantial to support COCs maturation, and as a consequence, it promotes proper embryonic development. Due to this fact, IO+DHEA inhibition significantly decreased embryo culture efficiency. Most of the changes observed within the oocytes after IVM were similar both in the IO+DHEA and the ETOMOXIR experimental groups. Interestingly, substantial differences in the reaction to the two inhibition systems applied were observed in the cumulus cells (e.g., mRNA relative level or metabolome enrichment analysis; summarized data are shown in Table 3). Moreover, etomoxir treatment significantly changed consumption/secretion of several fatty acids from/to the maturation medium. We suggest that glucose metabolism is essential for COC, and the consequences of its inhibition may not be fully substituted by the products of lipid energy pathways.

Numerous factors have an impact on oocyte quality and the ovarian follicular environment, in which the oocyte grows and matures. What is important is that the prolonged effect of the oocyte’s maturation environment is detected also during further stages of embryo development, resulting, for example, in the delayed progression of meiosis and lower fertilization, cleavage, and blastocyst rates [14,24]. Our study showed that inhibition of glucose metabolism at the time of IVM caused a significant decrease in both parameters. Since the negative impact of glucose inhibition has already been seen during the first cleavage division, this indicates a substantial reduction in the developmental competence of maturing oocytes. Glucose is the main source of energy for COC, although fatty acid oxidation may be an alternative and more efficient energy pathway [25]. Inhibitors used in the present experiment blocked glycolysis as well as PPP. It has been previously reviewed that COCs of reduced developmental competence are characterized by low glycolytic activity [7]. PPP is also substantial for proper nuclear maturation of oocytes since it was shown that it is involved in purine synthesis [26]. Therefore, our results support the statement of the importance of glucose metabolism during oocyte development and its impact on embryo development. To our knowledge, this is the first published evidence of the impact of two energy metabolism inhibitors during IVM on in vitro development of bovine embryos.

Many aspects of oocyte maturation (changes within the cytoplasm, along with molecular and nuclear modifications) are crucial for successful fertilization and proper embryo development [27]. It is due to the fact that the oocyte during its growth and maturation accumulates transcripts, proteins, and energy sources, which support further embryo metabolism, especially during the first hours/days, when the embryonic genome is inactive. We observed numerous changes in the experimental groups when compared to control (e.g., lower LD count and lipid content in LD, higher ATP level, changes in metabolic pathways activities); however, surprisingly, most of them were similar in both inhibitory systems. 

In both experimental groups, we have noted a significant increase in the ATP content in the oocytes subjected to the IVM. Therefore, the presence of energy metabolism inhibitors may have increased the general production of energy in the oocytes. It opposes the results of previous studies, in which treatment with either DHEA or IO significantly reduced the ATP level in mouse oocytes [11]. In the literature, there are no similar experiments performed on cattle, but it was shown that mouse and bovine oocytes exhibit different cellular responses to energy inhibitors [25]. Moreover, ATP provides only a limited indication of the oocyte’s energy supply because the energy status of a cell is also related to the concentrations of ADP and AMP. Additionally, some experimental evidence suggests that the oocytes are able to generate some ATP via the adenosine salvage pathway, a two-step process of AMP phosphorylation to the ATP [8]. Considering the current state of knowledge in the field, further studies on ATP production in described inhibitory systems are suggested.

Other aspects of our experiment suggest possible disturbances in the protection mechanism against the reactive oxygen species in oocytes. Glutathione is a key antioxidant required for proper oocyte maturation and early embryo development [8]. Products of PPP include NADPH, which is utilized during the reduction of glutathione. In our experiment, surprisingly, inhibition of both glucose and lipids metabolism did not affect glutathione content in oocytes during in vitro maturation. Different observations were made for mouse oocytes, in which the total GSH level decreased significantly after maturation in the presence of DHEA or IO [11]. In this experiment, however, additional supplements to the maturation medium were applied (glucose, lactate), which could substantially impact the final result. Additionally, in our studies, also the gene expression data in cumulus cells showed an unaffected mRNA level of the *TXNRD1* gene, the product of which is involved in the protection against oxidative stress. Therefore, we conclude that COCs subjected to tested inhibition conditions did not suffer oxidative stress.

Interestingly, in oocytes of both experimental groups, total lipid content in lipid droplets decreased, whereas LD count was lower only in IO+DHEA experimental group. It has been shown that during bovine oocyte maturation, the number of lipid droplets significantly increases [14]. This parameter may be affected by the maturation conditions and other external factors such as the mother’s diet and lactation status [28]. In the present experiment, lower lipid droplets content in both experimental groups indicates compromised accumulation of fatty acids during IVM in both inhibition systems, which may negatively affect further development. It confirms previous findings, in which lower lipid content after etomoxir treatment was observed in mouse oocytes [29]. Changes observed in LD are supported by metabolome enrichment analysis, which showed lower fatty acid biosynthesis metabolism in both experimental groups. It consequently indicates impaired lipid metabolism both after glucose and fatty acids metabolism inhibition. On the contrary, analyzing the lipidome of oocytes, we have noticed that except for GPL-diether and LPE lipids, the levels of other lipid groups were not significantly reduced when compared to the control, due to high sample variability. Similar results were obtained by [25], whose studies did not show changes in lipid content within oocytes matured in the presence of etomoxir. As for glucose inhibition, a lower lipid content was described in GV oocytes of mice fed with DHEA (inhibitor of PPP pathway) [30]. The PPP leads to the production of NADPH, and lipid synthesis is the process that requires NADPH. Authors suggest that PPP inhibition may negatively affect lipid synthesis by limiting NADPH supply. Hence, it is not clear how to interpret lipid content changes in the oocytes in the present experiment. It is possible that lipid reservoirs within the oocyte are not submitted to substantial changes shortly after 24h treatment of inhibitors and they need longer treatment for visible reaction.

Since only minor differences between the oocytes of both experimental groups were observed, an effect should be noticed within the cumulus cells, which play a crucial role in the energy metabolism of COCs. CCs absorb and convert glucose, transferring further metabolites to the oocyte. With regard to lipid metabolism, CCs are also crucial, accumulating lipids from the environment as a reservoir, on the one hand, and protecting from the lipotoxic effect of selected fatty acids, on the other hand [17]. Therefore, changes in the metabolism of CCs may affect the oocyte and consequently influence embryonic developmental competence. In our experiment, surprisingly, the total lipid content in LD remained constant, whereas the LD number decreased similarly in both experimental groups. It is in agreement with [31], who described that bovine cumulus cells contained fewer lipid droplets after maturation with ETOMOXIR in relation to the maturation control group. In LD, lipids are accumulated in the form of TAGs, the concentration of which also did not differ according to lipidome analysis in experimental groups vs. control. We also observed that TAGs were a dominant group of lipids both in oocytes and cumulus cells, which was previously described in oocytes by [32]. It was also noticed, especially in the cumulus cells, that both inhibition systems decreased the percentage contribution of TAGs within all lipid groups detected. This could indicate either reduction in TAGs synthesis or an increase in TAGs hydrolysis, as was suggested by [16]. FAs that were not transformed into TAGs might have been transported into vacuoles. This observation was confirmed in the ETOMIXIR group, in which a higher expression of the *CD36* gene (one of the main FA transporters), was detected. However, despite the metabolic disorders, cumulus cells of the ETOMOXIR group were able to keep the lipid storage status constant, thus preventing any adverse effects on the development of the nascent embryo.

In CCs, differences were observed between the experimental groups with regard to the expression of several genes involved in glucose (*PDHA1, SLC2A1*) and FA metabolism (*FASN, PLIN2, CD36*). *PDHA1* and *SLC2A1* genes were not affected in the IO+DHEA group; however, both were significantly downregulated in the ETOMOXIR group. *PDHA1* is a gene encoding an enzymatic subunit of pyruvate dehydrogenase complex that catalyzes the first step in the oxidative metabolism of pyruvate. The product of the *SLC2A1* is a gene that facilitates the transport of glucose across the plasma membranes. The results indicate that FAO inhibition may negatively affect substantial pathways of glucose metabolism in cumulus cells. It confirms the results of [25], who suggested that an inhibitory dose of etomoxir limits the proper glycolysis in CCs.

Interestingly, in both experimental groups, a lower transcript level of the *PFKP* gene was observed. PFKP is a key enzyme involved in the regulation of glycolysis; therefore, cumulus cells downregulated their glycolysis in both inhibitory conditions. Since glycolysis in cumulus cells is regulated by the paracrine factor(s) secreted by oocytes [33], the observed downregulation might be controlled by the oocyte.

With regard to genes involved in fatty acids metabolism, the most interesting differences revealed the following genes: *FASN* (involved in lipogenesis), *PLIN2* (involved in FA storage), and *CD36* (responsible for FA incorporation from the extracellular environment). *FASN* was downregulated in the ETOMOXIR group. *PLIN2* was downregulated in the IO+DHEA group but upregulated in the ETOMOXIR group. *CD36* gene was strongly upregulated only in the ETOMOXIR group. It was previously shown that FAO inhibition affects the expression of genes involved both in glucose and FA metabolism in cumulus cells [25,32]. We suggest that IO+DHEA treatment may reduce FA storage, whereas ETOMOXIR reduces FA synthesis along with increased FA collection from external sources (follicular fluid, maturation medium) as well as FA storage. It is confirmed by our metabolomic analysis of maturation medium, in which significantly higher uptake of decanoic, linoleic, and arachidic acids after ETOMOXIR treatment was observed. 

The most noteworthy results of the present study were obtained from metabolome enrichment analysis, in which etomoxir treatment did not affect any metabolic pathways in CC, whereas glucose metabolism inhibition upregulated ketone body and β-alanine metabolisms. According to [34], when the level of serum-free FA is raised in the organism, proportionately more free FA is converted to the ketone bodies (the metabolism of which produces less ATP), and less is oxidized via the citric acid cycle. The partition of acetyl–CoA between the ketogenic pathway and oxidation to CO_2_ is regulated in order to keep the constant status of the total free energy captured in ATP. Therefore, higher ketone body metabolism in the IO+DHEA group may indicate a higher level of free FA in cumulus cells. Although ketone utilization is characteristic for cellular metabolism in dairy cattle, negative energy balance condition in dairy cattle is characterized by elevated levels of NEFAs and ketone bodies in the blood serum [35]. According to [36], cumulus cells treated with one of the major ketone bodies—β-hydroxybutyrate (BOHB)—showed overexpression of the *PPARA* (peroxisome proliferator activated receptor α) gene, a master regulator of lipid metabolism. The authors suggest that cumulus cells obtained from cows in ketosis stimulate lipid metabolism and pose an ability to reprogram in order to utilize alternative sources of energy. Therefore, the upregulation of the ketone bodies metabolism in the IO+DHEA treated cumulus cells may also suggest their efforts toward activation of lipids metabolism; however, the final effect of these efforts was not observed. Moreover, gene expression results showed either lack of effect or downregulation of expression of genes involved in lipid metabolism. In our opinion, this suggests that cumulus cells, being under glucose metabolism inhibitory conditions, may undertake attempts to switch into the production of energy from lipid sources. However, due to unknown factors, this process fails, resulting in reduced embryo development.

Another metabolic pathway significantly upregulated in the IO+DHEA treated cumulus cells was β-alanine metabolism. β-alanine is a naturally occurring nonessential β amino acid, which is formed in organisms by different metabolic pathways. In human skeletal muscles, culture with β-alanine promoted lipid oxidation [37]. However, to date, there is no information on the role of this pathway in oocytes or cumulus cell metabolism.

## 5. Limitations

The present experiment aimed to investigate how the changes in the basic metabolism during in vitro maturation affect COCs and further embryonic development and to validate the possible plasticity in choosing the energy source. Since glucose and FA are the most readily utilized energy sources, the experimental design concentrated on selective inhibition of either of the two energy production pathways. This model has its limitations since it does not allow to evaluate precisely the importance of glycolysis or PPP pathways (blocked by two inhibitors, which are used together in the IO+DHEA experimental group) in COC energy metabolism balance. Anaerobic glycolysis is a major pathway for glucose metabolism in the mammalian ovarian cells, whereas the PPP pathway is crucial for cytoplasmic integrity and redox state, and it takes part in RNA/DNA synthesis [17]. It is, however, unclear which pathway might be the leader in the hypothesized crosstalk with lipid metabolism within the COC. Conversely, maturation in a medium supplemented with inhibitors of both glucose and FA metabolism could indicate the general importance of energy metabolism in the COC maturation process. This approach would show how COCs recompense crucial deficits of energy sources, and how it affects further embryo development. Despite the described limitations, the present data do provide solid grounds for further experiments on the importance of energy metabolism during COC maturation.

## 6. Conclusions

The obtained results indicate that glucose metabolism during in vitro maturation is substantial for the oocyte since only IO+DHEA inhibition significantly decreased embryo culture efficiency determined by reduced cleavage, blastocyst, and hatched blastocyst rate. Numerous changes were observed in the oocytes of the experimental groups when compared to the control group. The differences included lower LD count and lipid content in LD, higher ATP level, and changes in metabolic pathways activities; yet most of them were similar in both of the studied inhibitory systems. The most noteworthy differences in reaction on either IO+DHEA or etomoxir were observed in CCs (mRNA relative level or metabolic pathways disorders). Ketone body metabolism upregulation in CCs of the IO+DHEA group indicates possible efforts of the cells to switch into the lipid utilization, which was not sufficient in the result for proper embryo development. On the contrary, etomoxir treatment during IVM did not affect further embryo development, probably due to undisturbed metabolic pathways in CC. We suggest that glucose metabolism is substantial for proper COC maturation, whereas disturbances in fatty acid metabolism might be overcome, probably due to the protective role of cumulus cells. Therefore, our studies indicate that both of the energy pathways are not interchangeably alternatives in bovine COCs. Almost 20 years ago, the study in [38] suggested possible glucose–fatty acid metabolism homeostasis in the cells. Later, an orchestrated balance between glucose and fatty acid metabolism in mouse COCs was described [32]. Based on our results, we suggest that the plasticity of COCs with regard to the energy source is unlikely, which, in our opinion, is the crucial conclusion of the present experiment.

## Figures and Tables

**Table 1:** The sequences of primers and probes, their efficiency, and the length of the products obtained from mRNA gene expression experiment. Selected genes were analyzed in cumulus cells. F – forward primer sequence, R – reverse primer sequence, P – probe sequence.

Gene		Sequences of Primers (F, R) and TaqMan Probes (P)	Length (bp)	Efficiency
*ACACA* Acetyl-CoA carboxylase α Gene ID: 281590	F R P	GGGAACCGTGAAGGCCTA TGGACCAAGCTGCGGAT F–ATCACCGAGCGGACGCCGT–Q	159	1.942
*CPT1B* Carnitine palmitoyltransferase 1B Gene ID: 509459	F R P	AAGAGATCAAGCCTGTGATGGC GGATGCGAGTGGTGTTGAA F–CTGGGCCTCGTGCCCATGT–Q	84	1.944
*PLIN2* Perilipin 2 Gene ID: 280981	F R P	GAGTGGAAGAGAAGCATCGG GTGACTCAATGTGCTCAGCA F–CGATGATACAGATGAATCCCACTGTG–Q	67	1.822
*TXNRD1* Thioredoxin reductase 1 Gene ID: 282388	F R P	GCCAAGGAGGCAGCCA GTTCCTCCAAGACCCCATCTA F–TCCAAGAGGGGTTGGAGTGACAA–Q	92	1.934
*FASN* Fatty acid synthase Gene ID: 281152	F R P	CTGCCGAAGACAGGGATTG CTGGTATACCTTCCCGCTCG F–AGTGCGGCTTCTGGAAGCTTCC–Q	108	1.907
*CD36* Gene ID: 281052	F R P	AAAAATTGAAGCATTGAAGAATCTGA CCCAGTCACTTGATTTCTGAACA F–CCATTGGTGATGAGAAGGCGGA–Q	118	1.919
*PFKP* Phosphofructokinase Gene ID: 507119	F R P	CGGGACCTGCAGTCCAAC CTGCAGCTCTCGTTCCTGAG F–CATCTTCTCCGTCAGGTGCTCCA–Q	89	1.894
*LDHA* Lactate dehydrogenase A Gene ID: 281274	F R P	TGACTCTAGTGTGCCTGTATGG GATCACCTCATAAGCACTGTCAA F–TGAATGTTGCTGGTGTCTCCCTGA–Q	145	1.895
*PDHA1* Pyruvate dehydrogenase Gene ID: 407109	F R P	TGCAGAGCTTACAGGACGAA TCCCAGGGGCACCTGA F–CACGATGCCATTGCCTCCGT–Q	118	1.948
*SLC2A1* Solute carrier family 2 member 1 Gene ID: 282356	F R P	GCTACAACACTGGAGTCATCAACG CGGAGAAGGAACCAATCATGC F–AGTTCTACAACCAGACGTGGGTCCAGC–Q	168	1.882
*G6PDH* Glucose-6-phosphate dehydrogenase Gene ID: 281179	F R P	CACCATCTGGTGGCTGTTC TTCTCCTCCGGGGTAGCT F–ATCCGCAAGCAGAGCGAGCC–Q	135	1.852
*bCS* bos Citrate Synthase Gene ID: 280682	F R P	ATCACTGTGGACATGATGTATGG CTCTAAAACGGATGCCCTCA F–ATCCCTTCATGCCTCGCATGCC–Q	97	1.932
*GAPDH* Glyceraldehyde-3 phosphate dehydrogenase Gene ID: 281181	F R P	ACCCTCAAGATTGTCAGCAA GCGTGGACAGTGGTCATAAG F–CCTCCACGATGCCAAAGTGGTC–Q	113	1.975
*YWHAZ* Tyrosine 3-monooxygenase Gene ID: 287022	F R P	TGAACTCCCCTGAGAAAGCCT ATCCGATGTCCACAATGTCAAG F–AGCATTTGATGAAGCCATTGCTGAACTTGA–Q	149	1.918

**Table 2:** Fatty-acid-concentration changes detected in the maturation medium (mean values). The positive values of concentration indicate higher concentration after IVM when compared to the medium before IVM (COCs secreted selected FA). The negative values of concentration indicate lower concentration after IVM when compared to the medium before IVM (COCs collected selected FA). Values are significantly different when * *p* ≤ 0.05 or ** *p* ≤ 0.01.

	CONTROL (Post-IVM Medium vs. Pre-IVM Medium)	IO+DHEA			ETOMOXIR		
		Post-IVM Medium vs. Pre-IVM Medium	p (IO+DHEA vs. CONTROL)	IO+DHEA vs. CONTROL	Post-IVM Medium vs. Pre-IVM Medium	*p* (ETOMOXIR vs. CONTROL)	ETOMOXIR vs. CONTROL
Caprylic/ Octanoid acid (C8:0)	−3.45 × 10^−5^	−3.34 × 10^−5^	0.4277		−7.39 × 10^−5^	0.0353 *	Higher uptake
Caprinic/ Decanoic acid (C10:0)	3.68 × 10^−5^	4.70 × 10^−5^	0.4832		−1.63 × 10^−5^	0.0002 **	Higher uptake
Lauric/ Dodecanoic acid (C12:0)	−2.84 × 10^−5^	3.87 × 10^−5^	0.0715		3.43 × 10^−6^	0.0984	
Myristic /Tetradecanoic acid (C14:0)	−2.05 × 10^−4^	3.33 × 10^−4^	0.0534		5.60 × 10^−4^	0.009 **	Higher secretion
Pentadecanoic acid (C15:1)	−1.38 × 10^−4^	2.35 × 10^−5^	0.0047 **	Higher secretion	1.75 × 10^−5^	0.0131 *	Higher secretion
Palmitic acid (C16:0)	1.67 × 10^−3^	1.73 × 10^−3^	0.4267		6.06 × 10^−4^	0.0216 *	Lower secretion
Stearic acid (C18:0)	1.87 × 10^−3^	2.10 × 10^−3^	0.3335		8.40 × 10^−4^	0.1159	
Oleic acid (C18:1n9)	3.04 × 10^−5^	3.48 × 10^−5^	0.3174		−3.02 × 10^−6^	0.0708	
Linoleic acid (C18:2)	5.82 × 10^−6^	−8.75 × 10^−6^	0.4747		−2.06 × 10^−5^	0.0341 *	Higher uptake
Arachidic/ Eicosanoic acid (C20:0)	−8.66 × 10^−6^	−6.20 × 10^−6^	0.3878		−2.84 × 10^−5^	0.0292 *	Higher uptake

**Table 3:** Summary of the most important and significant results of the experiments observed within the cumulus–oocyte complex. The table shows the comparison of experimental groups vs. control. ↓—significant downregulation of the parameter; ↑—significant upregulation of the parameter; =—no significant change of the parameter.

Parameters	IO + DHEA		ETOMOXIR	
	Oocytes	Cumulus Cells	Oocytes	Cumulus Cells
**Lipid Droplets**				
LD count	↓	↓	=	↓
LD average size (µM)	=	↑	=	=
total lipid content	↓	=	↓	=
**Gene Expression**				
PFKP		↓		↓
PDHA1		=		↓
SLC2A1		=		↓
ACACA		↓		↓
CPT1B		↓		↓
FASN		=		↓
PLIN2		↓		↑
CD36		=		↑
**ATP**	↑		↑	
**Glutathione**	=		=	
**Metabolome Enrichment Analysis**				
fatty acid biosynthesis	↓	=	↓	
ketone body metabolism	=	↑		=
β-alanine metabolism	=	↑		=
**Lipidome Analysis**				
concentration of GPL-diether	↑	↑	=	=
concentration of other lipid classes	=	=	=	=
% distribution of lipid classes	=	=	=	=

## Data Availability

The data presented in this study are openly available.

## Supplementary Materials

The following are available online at https://www.mdpi.com/article/10.3390/genes12060838/s1, Supplementary File S1.
