# Supplementary material for: Response of Bovine Cumulus–Oocytes Complexes to Energy Pathway Inhibition during In Vitro Maturation
Source: Genes (Basel). 2021 May 29;12(6):838. doi: 10.3390/genes12060838 (PMC8228821; doi:10.3390/genes12060838)
Supplement: Supplementary file 1 [file genes-12-00838-s001.zip › genes-1173209-supplementary.pdf]

The results of Primer-BLAST analysis with the bioinformatics tool: https://www.ncbi.nlm.nih.gov/tools/primer-blast/index.cgi

Primer pair for ACACA gene

	Sequence (5'->3')	Length	Tm	GC%	Self complementarity
Forward primer	GGGAACCGTGAAGGCCTA	18	58.60	61.11	6.00
Reverse primer	TGGACCAAGCTGCGGAT	17	58.48	58.82	5.00

>NM_174224.2 Bos taurus acetyl-CoA carboxylase alpha (ACACA), mRNA

```
product length = 159
Forward primer  1     GGGAACCGTGAAGGCCTA  18
Template        7108  ..................  7125

Reverse primer  1     TGGACCAAGCTGCGGAT  17
Template        7266  .................  7250
```

Primer pair for CPT1B

	Sequence (5'->3')	Length	Tm	GC%	Self complementarity
Forward primer	AAGAGATCAAGCCTGTGATGGC	22	60.68	50.00	7.00
Reverse primer	GGATGCGAGTGGTGTTGAA	19	58.07	52.63	3.00

>NM_001034349.2 Bos taurus carnitine palmitoyltransferase 1B (CPT1B), mRNA

```
product length = 84
Forward primer  1     AAGAGATCAAGCCTGTGATGGC  22
Template        1076  ......................  1097

Reverse primer  1     GGATGCGAGTGGTGTTGAA  19
Template        1159  ...................  1141
```

Primer pair for PLIN2 gene

	Sequence (5'->3')	Length	Tm	GC%	Self complementarity
**Forward primer**	GAGTGGAAGAGAAGCATCGG	20	58.07	55.00	4.00
**Reverse primer**	GTGACTCAATGTGCTCAGCA	20	58.48	50.00	7.00

>NM_173980.2 Bos taurus perilipin 2 (PLIN2), mRNA

```
product length = 67
Forward primer  1    GAGTGGAAGAGAAGCATCGG  20
Template        915  ....................  934

Reverse primer  1    GTGACTCAATGTGCTCAGCA  20
Template        981  ....................  962
```

Primer pair for TXNRD1 gene

	Sequence (5'->3')	Length	Tm	GC%	Self complementarity
**Forward primer**	GCCAAGGAGGCAGCCA	16	59.16	68.75	5.00
**Reverse primer**	GTTCCTCCAAGACCCCATCTA	21	58.52	52.38	3.00
**Products on target templates**					

>NM_174625.5 Bos taurus thioredoxin reductase 1 (TXNRD1), mRNA

```
product length = 92
Forward primer  1    GCCAAGGAGGCAGCCA  16
Template        299  ................  314

Reverse primer  1    GTTCCTCCAAGACCCCATCTA  21
Template        390  .....................  370
```

Primer pair for FASN gene

	Sequence (5'->3')	Length	Tm	GC%	Self complementarity
Forward primer	CTGCCGAAGACAGGGATTG	19	58.23	57.89	3.00
Reverse primer	CTGGTATACCTTCCCGCTCG	20	59.69	60.00	8.00

>NM_001012669.1 Bos taurus fatty acid synthase (FASN), mRNA

```
product length = 108
Forward primer  1      CTGCCGAAGACAGGGATTG  19
Template        2863   ...................  2881

Reverse primer  1      CTGGTATACCTTCCCGCTCG  20
Template        2970   ....................  2951
```

Primer pair for CD36 gene

	Sequence (5'->3')	Length	Tm	GC%	Self complementarity
Forward primer	AAAAATTGAAGCATTGAAGAATCTGA	26	56.88	26.92	5.00
Reverse primer	CCCAGTCACTTGATTTCTGAACA	23	58.86	43.48	5.00

>NM_001278621.1 Bos taurus CD36 molecule (CD36), transcript variant 1, mRNA

```
product length = 118
Forward primer  1      AAAAATTGAAGCATTGAAGAATCTGA  26
Template        1604   ..........................  1629

Reverse primer  1      CCCAGTCACTTGATTTCTGAACA  23
Template        1721   .......................  1699
```

>NM_174010.3 Bos taurus CD36 molecule (CD36), transcript variant 2, mRNA

```
product length = 118
```

```
Forward primer  1     AAAAATTGAAGCATTGAAGAATCTGA  26
Template        1446  ..........................  1471

Reverse primer  1     CCCAGTCACTTGATTTCTGAACA  23
Template        1563  .......................  1541
```

Primer pair for PFKP gene

	Sequence (5'->3')	Length	Tm	GC%	Self complementarity
Forward primer	CGGGACCTGCAGTCCAAC	18	60.36	66.67	8.00
Reverse primer	CTGCAGCTCTCGTTCCTGAG	20	60.46	60.00	6.00

Products on target templates

>NM_001193220.3 Bos taurus phosphofructokinase, platelet (PFKP), mRNA

```
product length = 89
Forward primer  1     CGGGACCTGCAGTCCAAC  18
Template        1893  ..................  1910

Reverse primer  1     CTGCAGCTCTCGTTCCTGAG  20
Template        1981  ....................  1962
```

Primer pair for LDHA gene

	Sequence (5'->3')	Length	Tm	GC%	Self complementarity
Forward primer	TGACTCTAGTGTGCCTGTATGG	22	59.24	50.00	6.00
Reverse primer	GATCACCTCATAAGCACTGTCAA	23	58.49	43.48	4.00

>NM_174099.2 Bos taurus lactate dehydrogenase A (LDHA), mRNA

```
product length = 145
Forward primer  1    TGACTCTAGTGTGCCTGTATGG  22
Template        740  ......................  761
```

```
Reverse primer  1    GATCACCTCATAAGCACTGTCAA  23
Template        884  .......................  862
```

Primer pair for PDHA1 gene

	Sequence (5'->3')	Length	Tm	GC%	Self complementarity
Forward primer	TGCAGAGCTTACAGGACGAA	20	59.03	50.00	4.00
Reverse primer	TCCCAGGGGCACCTGA	16	58.93	68.75	8.00

>NM_001101046.2 Bos taurus pyruvate dehydrogenase E1 subunit alpha 1 (PDHA1), mRNA; nuclear gene for mitochondrial product

```
product length = 118
Forward primer  1    TGCAGAGCTTACAGGACGAA  20
Template        509  ....................  528

Reverse primer  1    TCCCAGGGGCACCTGA  16
Template        626  ................  611
```

Primer pair for SLC2A1 gene

	Sequence (5'->3')	Length	Tm	GC%	Self complementarity
Forward primer	GCTACAACACTGGAGTCATCAACG	24	61.90	50.00	4.00
Reverse primer	CGGAGAAGGAACCAATCATGC	21	59.33	52.38	4.00

Products on target templates

>NM_174602.2 Bos taurus solute carrier family 2 member 1 (SLC2A1), mRNA

```
product length = 168
Forward primer  1    GCTACAACACTGGAGTCATCAACG  24
Template        250  ........................  273

Reverse primer  1    CGGAGAAGGAACCAATCATGC  21
Template        417  .....................  397
```

Primer pair for G6PDH gene

	Sequence (5'->3')	Length	Tm	GC%	Self complementarity
Forward primer	CACCATCTGGTGGCTGTTC	19	58.44	57.89	8.00
Reverse primer	TTCTCCTCCGGGGTAGCT	18	58.89	61.11	6.00

Products on target templates

>NM_001244135.2 Bos taurus glucose-6-phosphate dehydrogenase (G6PD), mRNA

```
product length = 135
Forward primer  1    CACCATCTGGTGGCTGTTC  19
Template        240  ...................  258

Reverse primer  1    TTCTCCTCCGGGGTAGCT  18
Template        374  ..................  357
```

Primer pair for bCS gene

	Sequence (5'->3')	Length	Tm	GC%	Self complementarity
Forward primer	ATCACTGTGGACATGATGTATGG	23	58.29	43.48	8.00
Reverse primer	CTCTAAAACGGATGCCCTCA	20	57.31	50.00	3.00

>NM_001044721.1 Bos taurus citrate synthase (CS), mRNA; nuclear gene for mitochondrial product

```
product length = 97
Forward primer  1    ATCACTGTGGACATGATGTATGG  23
Template        298  .......................  320

Reverse primer  1    CTCTAAAACGGATGCCCTCA  20
Template        394  ....................  375
```

Primer pair for GAPDH gene

	Sequence (5'->3')	Length	Tm	GC%	Self complementarity
Forward primer	ACCCTCAAGATTGTCAGCAA	20	57.04	45.00	4.00
Reverse primer	GCGTGGACAGTGGTCATAAG	20	58.64	55.00	3.00

Products on target templates

>NM_001034034.2 Bos taurus glyceraldehyde-3-phosphate dehydrogenase (GAPDH), mRNA

```
product length = 113
Forward primer  1    ACCCTCAAGATTGTCAGCAA  20
Template        492  ....................  511

Reverse primer  1    GCGTGGACAGTGGTCATAAG  20
Template        604  ....................  585
```

Primer pair for YWHAZ gene

	Sequence (5'->3')	Length	Tm	GC%	Self complementarity
Forward primer	TGAACTCCCCTGAGAAAGCCT	21	60.77	52.38	3.00
Reverse primer	ATCCGATGTCCACAATGTCAAG	22	58.73	45.45	5.00

>NM_174814.2 Bos taurus tyrosine 3-monooxygenase/tryptophan 5-monooxygenase activation protein zeta (YWHAZ), mRNA

```
product length = 149
Forward primer  1    TGAACTCCCCTGAGAAAGCCT  21
Template        598  .....................  618

Reverse primer  1    ATCCGATGTCCACAATGTCAAG  22
Template        746  ......................  725
```
